# The Effect of Potassium Canrenoate (Mineralocorticoid Receptor Antagonist) on the Markers of Inflammation in the Treatment of COVID-19 Pneumonia and Fibrosis—A Secondary Analysis of Randomized Placebo-Controlled Clinical Trial

**DOI:** 10.3390/ijms241814247

**Published:** 2023-09-18

**Authors:** Igor Karolak, Rafał Hrynkiewicz, Paulina Niedźwiedzka-Rystwej, Kacper Lechowicz, Jerzy Sieńko, Aleksandra Szylińska, Wojciech Dabrowski, Katarzyna Kotfis

**Affiliations:** 1 Department of Anesthesiology, Intensive Therapy and Acute Intoxications, Pomeranian Medical University in Szczecin, 70-111 Szczecin, Poland; igor.karolak@gmail.com (I.K.); kacper.lechowicz@gmail.com (K.L.); 2 Institute of Biology, University of Szczecin, 71-412 Szczecin, Poland; rafal.hrynkiewicz@usz.edu.pl (R.H.); paulina.niedzwiedzka-rystwej@usz.edu.pl (P.N.-R.); 3 Institute of Physical Culture Sciences, University of Szczecin, 70-453 Szczecin, Poland; jsien@poczta.onet.pl; 4 Department of Medical Rehabilitation and Clinical Physiotherapy, Pomeranian Medical University in Szczecin, 71-210 Szczecin, Poland; aleksandra.szylinska@gmail.com; 5 Department of Anaesthesiology, Intensive Care Medical University of Lublin, 20-059 Lublin, Poland; w.dabrowski5@gmail.com

**Keywords:** SARS-CoV-2, COVID-19, potassium canrenoate, inflammatory markers, cytometry, cytokines, interleukin-6, fibrosis

## Abstract

In March 2020, the World Health Organization (WHO) announced a global pandemic of coronavirus disease 2019 (COVID-19) that presented mainly as an acute infection of the lower respiratory tract (pneumonia), with multiple long-term consequences, including lung fibrosis. The aim of this study was to evaluate the influence of potassium canrenoate on inflammatory markers in the treatment of COVID-19 pneumonia. A randomized clinical trial (RCT) of intravenous potassium canrenoate vs. placebo was performed between December 2020 and November 2021. This study is a secondary analysis of that RCT. In the final analysis, a total of 49 hospitalized patients were included (24 allocated to the potassium canrenoate group and 25 to the placebo group). Patients were assessed by serum testing and blood cell cytometry on day 1 and day 7 of the intervention. Age, sex, and body mass index were not significantly different between the placebo group and intervention group. Although there was a significantly higher rate of ischemic heart disease in the placebo group, rates of other preexisting comorbidities were not significantly different. There were no significant differences in the inflammatory parameters between the potassium canrenoate and placebo groups on day 1 and day 7. However, the intragroup comparisons using Wilcoxon’s test showed significant differences between day 1 and day 7. The CD3% for potassium canrenoate increased significantly between day 1 and day 7 (12.85 ± 9.46; 11.55 vs. 20.50 ± 14.40; 17.80; *p* = 0.022), while the change in the placebo group was not significant (15.66 ± 11.39; 12.65 vs. 21.16 ± 15.37; 16.40; *p* = 0.181). The IL-1ß total count [%] increased over time for both potassium canrenoate (0.68 ± 0.58; 0.45 vs. 1.27 ± 0.83; 1.20; *p* = 0.004) and placebo (0.61 ± 0.59; 0.40 vs. 1.16 ± 0.91; 1.00; *p* = 0.016). The TNF-α total count (%) decreased significantly between day 1 and day 7 for potassium canrenoate (0.54 ± 0.45; 0.40 vs. 0.25 ± 0.23; 0.10; *p* = 0.031), but not for placebo (0.53 ± 0.47; 0.35 vs. 0.26 ± 0.31; 0.20; *p* = 0.056). Interleukin-6 (pg/mL) showed a significant decrease between day 1 and day 7 for potassium canrenoate (64.97 ± 72.52; 41.00 vs. 24.20 ± 69.38; 5.30; *p* = 0.006), but not the placebo group. This RCT has shown that the administration of potassium canrenoate to patients with COVID-19-induced pneumonia may be associated with significant changes in certain inflammatory markers (interleukin-6, CD3%, TNF-α), potentially related to pulmonary fibrosis. Although some positive trends were observed in the potassium canrenoate group, none of these observations reached statistical significance. Any possible benefits from the use of potassium canrenoate as an anti-inflammatory or antifibrotic drug in COVID-19 patients require further investigation.

## 1. Introduction

In March 2020, following the emergence of the SARS-CoV-2 virus initially identified in December 2019, the World Health Organization (WHO) declared a worldwide pandemic of coronavirus disease 2019 (COVID-19). This disease predominantly manifested as an acute infection affecting the lower respiratory tract, often presenting as pneumonia [1,2]. Since then, clinical and scientific teams around the world have focused on finding appropriate vaccination and a cure for this specific type of pneumonia, but also on limiting multiple long-term consequences, including lung fibrosis [3]. According to data from the World Health Organization (WHO), in November 2022, the total number of COVID-19 cases globally exceeded 640 million [4]. Most of the cases manifest as mild viral illness; however, in many patients, pneumonia leads to respiratory failure and acute respiratory distress syndrome (ARDS). Non-respiratory symptoms are also present, e.g., of thrombotic origin (i.e., embolism), or neurological derangement (i.e., neuropathic pain, delirium, long-term cognitive disturbances) [5,6]. SARS-CoV-2 infection has long-term consequences, including muscle weakness and fatigue, sleep disorders, anxiety, and depression, as well as pulmonary fibrosis [7].

It has been shown that severe COVID-19 is associated with a significant derangement in proinflammatory cytokines, namely, IL-1β, IL-2, IL-6, IL-8, or TNF-α, often referred to as the “cytokine storm” [8,9,10,11,12]. Interleukins from the IL-1 group play and important role in the induction of uncontrolled immune response in COVID-19 [13]. In COVID-19, the level of interleukin 10 is elevated and should act as an immunosuppressant, quite contrary to early pathological IL-10 elevation, which contributes to COVID-19 severity [14].

A prolonged increase in the level of IL-6 contributes to long COVID symptoms, so reducing IL-6 levels is particularly important during early COVID to prevent long-term sequelae [15].

The renin–angiotensin–aldosterone (RAA) system is a major player in the pathogenesis of COVID-19. The entry of SARS-CoV-2 virus into the cells is possible only as a result of the expression of the angiotensin-converting enzyme 2 (ACE2) receptor and the transmembrane serine protease 2 (TMPRSS2) [16]. In patients with moderate and severe COVID-19, the inflammatory response is induced by, e.g., the ACE2 pathway, where, due to the binding of the virus to ACE2, this enzyme does not convert angiotensin II to the anti-inflammatory angiotensin 1–7 [17,18,19,20,21]. In addition, high concentrations of ACE2 significantly increase the infectivity of the virus [16]. In the later stages of the disease, high concentrations of ACE2 may be protective against lung damage by increasing CCL-2, IL-8, and CCL-5 and reactive oxygen species due to angiotensin II/AT1R [19,21,22]. The amount of ACE2 is also affected by age and gender. Studies in rats have shown that ACE2 expression decreases with age, and in addition, in old age, the level of ACE2 in females was higher [23]. It has also been shown that in children, the amount of ACE2 decreases with age [24,25]. This may be the reason for the more severe course of COVID-19 in older men [26].

Under physiological conditions, the activator of the mineralocorticoid receptor (MR) is aldosterone. Its higher concentration can lead to hypertension, aggravation of cardiovascular diseases and also increased inflammation and tissue fibrosis [27,28].

There are studies suggesting a beneficial effect of mineralocorticoid receptor antagonists (MRAs) in the treatment of COVID-19 [29]. This group of drugs includes spironolactone and potassium canrenoate. The latter is the only clinically available form of mineralocorticoid receptor antagonists for intravenous use. These drugs are typically used to treat hypertension and congestive heart failure [30]. The protective effect of MRAs may be the result of ACE2 upregulation and antiandrogenic effects [31]. Spironolactone has been shown in preclinical studies to have antioxidant properties. It provides protection against oxidative stress and related damage through inhibition of free radical production and enhancement of antioxidant processes [32]. The use of spironolactone alleviated pneumonia associated with lipopolysaccharides and bleomycin [32]. Spironolactone might attenuate bleomycin-induced acute pulmonary injury and fibrosis by reducing the number of inflammatory cells in the alveoli, such as neutrophils, lymphocytes, macrophages or eosinophils [33]. Atalay et al. were able to show the effectiveness of spironolactone in the treatment of acute lung injury [34]. Additionally, Barut and coworkers analyzed the relationship between the use of spironolactone and lung damage associated with the reperfusion process of intestinal ischemia, suggesting that spironolactone decreased neutrophil infiltration, iNOS induction, oxidative stress, and histopathological injury [35]. The supply of spironolactone effected a reduction in neutrophil infiltration, oxidative stress and cellular damage, and also played a role in reducing the induction of nitric oxide synthase [29,33,36].

Potassium canrenoate is a mineralocorticoid receptor (MR) antagonist and a diuretic frequently used in practice for patients with chronic kidney disease, congestive heart failure or liver disease [37]. Moreover, potassium canrenoate mediates an anti-inflammatory response through the genomic effect through binding to the nuclear mineralocorticoid receptor and regulating the delay in gene transcription [38], manifesting over hours or days. Genomic effects can be extended due to a rapid nongenomic mechanism acting through membrane-bound receptors, the effect of which occurs within minutes [39].

Therefore, the purpose of the current study was to assess the influence of potassium canrenoate, an intravenous formula of mineralocorticoid receptor antagonist, on the change in inflammatory markers in patients with severe COVID-19-associated pneumonia.

## 2. Results

### 2.1. Baseline Patient Characteristics

There were no significant differences observed in age, gender, or body mass index between the placebo group and the intervention group. Table 1 presents the baseline characteristics and comorbidities of both study groups. However, it is noteworthy that the placebo group exhibited a notably higher prevalence of ischemic heart disease, which reached statistical significance. Rates of other preexisting comorbidities were not found to be significantly different between the two groups. Additionally, the Clinical Frailty Scale score was significantly higher in the placebo group compared to the intervention group, with a statistically meaningful difference noted (3.76 vs. 3.17, *p* = 0.034).

### 2.2. Basic Laboratory Tests

No statistically significant differences were observed between the groups in the results of basic initial laboratory tests conducted on day 1 or in the levels of serum inflammatory markers, which include C-reactive protein, procalcitonin, and interleukin-6, as detailed in Table 2.

There were no significant differences between the groups regarding the basic laboratory tests obtained on day 7 and serum inflammatory markers, including C-reactive protein and procalcitonin (Table 3).

### 2.3. Immunophenotype Analysis on Day 1 and Day 7

There were no significant differences between the groups in day 1 cell cytometry, including CD3+ blood cell or CD4+ lymphocyte percentage, IL-1ß, IL-2, TNF-α-positive cell percentage in general, lymphocytes specifically, or IL-6 level (Table 4).

There were no significant differences between the groups in control day 7 cell cytometry, including CD3+ blood cell or CD4+ lymphocyte percentage or IL-1ß, IL-2, TNF-α-positive cell percentage in general and on lymphocytes specifically, as well as IL-6 level (Table 5).

### 2.4. Intragroup Comparisons between Potassium Canrenoate and Placebo Groups on Day 1 versus Day 7

Intragroup comparisons using Wilcoxon’s test were performed to specify if the assessment of the dynamics of change between potassium canrenoate and placebo group or intragroup showed significant differences between day 1 and day 7. The results are shown in Table 4, Table 5 and Table 6.

**Figure 1:**
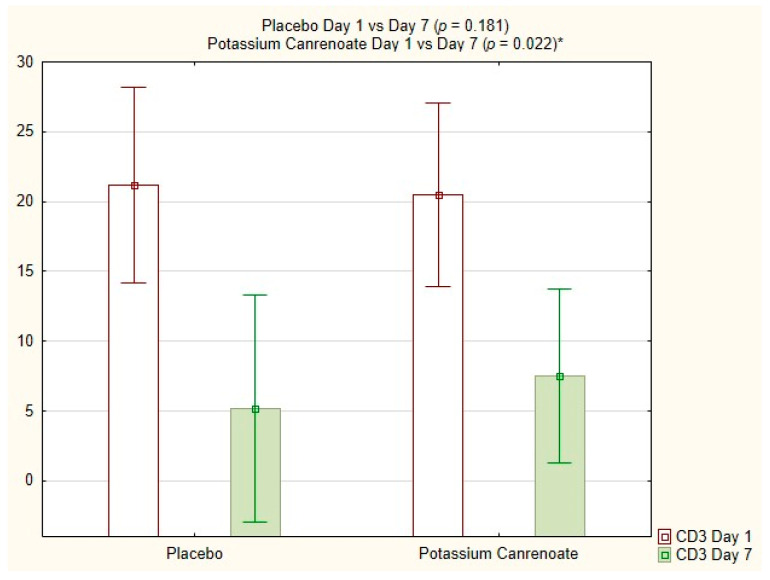
CD3% for potassium canrenoate vs. placebo between day 1 and day 7; *p*—statistical significance; * indicates statistical significance.

The CD3% for potassium canrenoate increased significantly between day 1 and day 7 (12.85 ± 9.46; 11.55 vs. 20.50 ± 14.40; 17.80; *p* = 0.022), as visible in Figure 1.

**Figure 2:**
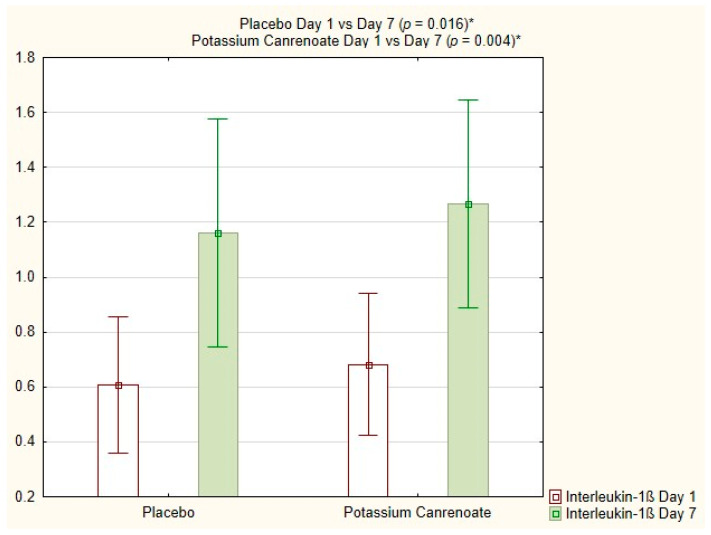
Interleukin-1ß total count [%] for potassium canrenoate vs. placebo between day 1 and day 7; *p*—statistical significance; * indicates statistical significance.

The interleukin-1ß total count (%) increased significantly over time for both potassium canrenoate (0.68 ± 0.58; 0.45 vs. 1.27 ± 0.83; 1.20; *p* = 0.004) and placebo (0.61 ± 0.59; 0.40 vs. 1.16 ± 0.91; 1.00; *p* = 0.016), as shown in Figure 2.

**Figure 3:**
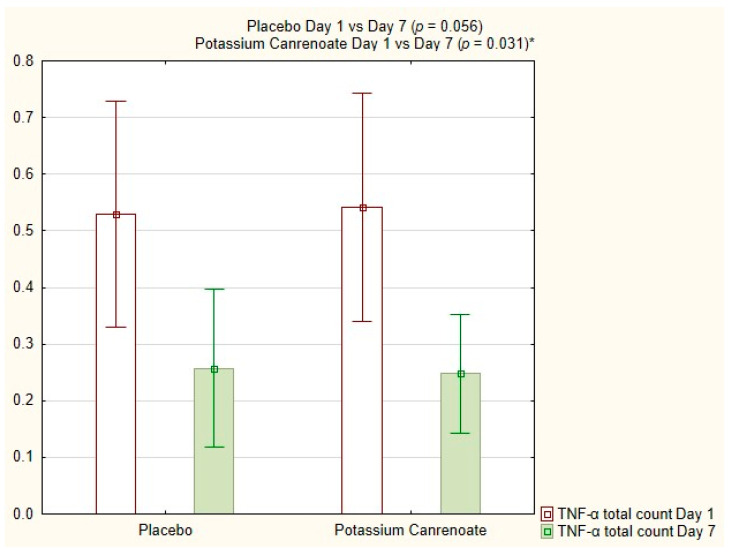
TNF-α total count [%] for potassium canrenoate vs. placebo between day 1 and day 7; *p*—statistical significance; * indicates statistical significance.

TNF-α total count (%) decreased significantly for potassium canrenoate between day 1 and day 7 (0.54 ± 0.45; 0.40 vs. 0.25 ± 0.23; 0.10; *p* = 0.031), but the changes noted for this parameter in the placebo group were not significant, as shown in Figure 3.

**Figure 4:**
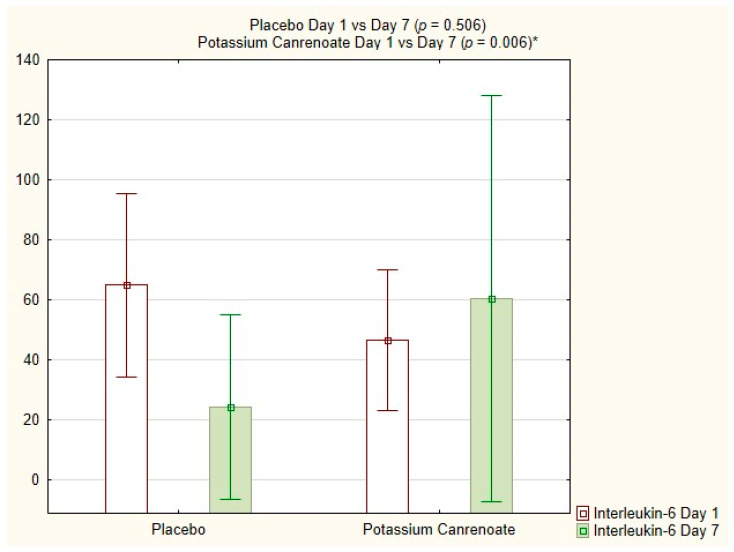
Interleukin-6 [pg/mL] for potassium canrenoate vs. placebo between day 1 and day 7; *p*—statistical significance; * indicates statistical significance.

Interleukin-6 (pg/mL) showed a significant decrease between day 1 and day 7 for potassium canrenoate (64.97 ± 72.52; 41.00 vs. 24.20 ± 69.38; 5.30; *p* = 0.006), which not visible in the placebo group, as shown in Figure 4.

## 3. Discussion

The increasing number of infections with the SARS-CoV-2 virus has created the need not only to treat the disease itself but also to develop a way to prevent long-term complications of infection deriving from immunological imbalance. In our study, we evaluated the effects of potassium canrenoate on inflammatory markers in the treatment of COVID-19.

There were no significant differences between the placebo and potassium canrenoate regarding CD3+ blood cell or CD4+ lymphocyte percentage, IL-1ß, IL-2, TNF-α positive cell percentage in general, or lymphocytes specifically on day 1 or on day 7. Similarly, the levels of Il-6 between the group did not differ in terms of statistical significance. However, the level of Il-6 between the placebo group and the intervention was higher in the intervention group on day 1 (46.68 ± 56.79 pg/mL vs. 64.97 ± 72.52 pg/mL, *p* = 0.332), but on day 7 after initiation of treatment, it was lower in the intervention group (60.56 ± 152.47 pg/mL vs. 24.20 ± 69.38 pg/mL, *p* = 0.317), although this was not statistically significant. The percentage of change between day 1 and day 7 was higher in the intervention group, with a 37.2% decrease in the intervention group compared to 29.7% increase in the placebo group. This may be a potential signal or trend of benefit in patients receiving potassium canrenoate, but this observation needs further evaluation in a larger RCT.

IL-6 is a cytokine characterized by its pro-inflammatory and pro-fibrotic properties. It plays a role in the development of pulmonary interstitial edema and triggers a severe inflammatory response by activating neutrophils and facilitating their accumulation at the injury site. This, in turn, leads to the release of both proteases and oxygen-free radicals, contributing to the overall inflammatory process [40,41]. Moreover, the level of Il-6 predicts the progression of disease to severe COVID-19 [40,41]. In the REMAP-CAP trial, the anti-IL-6 receptor antagonists tocilizumab and sarilumab improved the disease burden in critically ill patients with severe COVID-19 pneumonia [42] The importance of the biomarkers is indisputable in monitoring patients who suffer from COVID-19 towards evolution into pulmonary fibrosis.

While our study did not demonstrate a statistically significant benefit of administering additional potassium canrenoate therapy to patients with moderate to severe COVID-19 pneumonia, it is important to consider several potential explanations for this outcome. Spironolactone is one of the inhibitors of the renin–angiotensin–aldosterone (RAA) system, particularly preferred in COVID-19 [31]. There are studies suggesting the use of MRA as a prevention of fibrosis in coronavirus infections or other viral infections [43]. Pulmonary fibrosis is more commonly observed in patients who exhibit severe clinical symptoms, particularly in individuals with elevated levels of inflammatory markers [44]. Increased production of various cytokines and growth factors can lead to impaired healing processes and excessive scarring [45,46,47]. Inflammatory markers, e.g., CRP, IL6, have been proven to significantly increase with disease progression in COVID-19 patients and can be used to differentiate the severity of patients’ condition [48]. It has been shown only recently that in post-COVID-19 pulmonary fibrosis, extensive lung scarring occurs when two aspects are present: not only an increase in the level of transforming growth factor β (TGF-β), interleukin-6 (IL-6), and expression of matrix metalloproteinase 1 and 7 (MMP-1, MMP-7) but also collagen deposition at the site of lung injury [49]. In our study, we did not evaluate the level of TGF- β, MMP-1 or MMP-7, but the level of one of the above-mentioned cytokines, i.e., Il-6, was recorded and showed a larger decrease between day 1 and day 7 in the intervention group than in the placebo group. Therefore, apart from the cytokines evaluated by our group, other factors should be evaluated in future trials, including TGF-β, MMP-1 or MMP-7.

There have been reports suggesting a potential beneficial effect of potassium canrenoate in mitigating endothelial inflammation in individuals with SARS-CoV-2 infection [50]. Fels et al. also showed in in vitro studies that the use of spironolactone in COVID-19 attenuates the damage to the glycocalyx on renal tubular cells [51]. Several studies have suggested the therapeutic potential of spironolactone, as it has demonstrated the ability to significantly reduce the inflammatory response within the lungs following injury [33,36]. The BISCIUT study showed a significant reduction in fever duration with spironolactone and bromhexine [52]. Given its direct impact on lung endothelium and its indirect influence on modulating inflammatory responses and cytokine production, potassium canrenoate may offer potential benefits for patients suffering from advanced severe pneumonia due to COVID-19. Additionally, a study conducted by Umemura et al. suggested the anti-fibrosis potential of nintedanib in patients hospitalized in intensive care units, further emphasizing the potential therapeutic avenues for managing severe cases of COVID-19 [53]. Therefore, there is a need for further research involving patients requiring intensive care. Although the risk of fibrosis is higher in patients hospitalized in intensive care units, fibrosis has also been found in patients not undergoing mechanical ventilation [54,55].

The optimal timing and duration of potassium canrenoate intervention for COVID-19 pneumonia remain uncertain. In our study, patient inclusion was contingent upon having a blood oxygen saturation level below 94%, suggesting some degree of lung tissue damage. This criterion may have resulted in a delay in the timing of intervention. There is a legitimate concern that early immunomodulation could potentially suppress the host’s antiviral response and slow down viral clearance, whereas delayed immunomodulation may prove ineffective in the context of advanced acute lung injury [56]. However, some studies suggest that chronic use of MRA may be associated with a lower risk of COVID-19 infection, although without affecting the complications associated with this disease [57]. The results of a phase II randomized placebo-controlled trial of spironolactone and dexamethasone versus dexamethasone alone in hospitalized patients with confirmed COVID-19 recently reported by Wadhwa et al. showed significantly lower D-dimer levels on days 4 and 7 (*p* = 0.0004) and aldosterone at day 7 (*p* = 0.0075) in the SpiroDex group, but no significant difference in the time to recovery between SpiroDex and Dex groups. The authors concluded that low-dose oral spironolactone in addition to dexamethasone was safe, but phase III randomized controlled trials with a combined use of spironolactone and dexamethasone should be considered [58].

In our study, there was no significant increase in adverse events after administration of potassium canrenoate. Despite the reporting of some adverse events, such as hyperkaliemia, hyponatremia and hypovolemia, blood iron levels did not differ significantly between groups. Nevertheless, there was a trend towards hyperkalemia in the intervention group, which is the most common side effect of potassium canrenoate.

This study is not without limitations. Firstly, although the study was randomized, it was single-center, which may have contributed to the occurrence of biases. Secondly, it should be noted that the study was relatively small and the number of patients who completed the study was small. Thirdly, apart from the cytokines evaluated by our group (CD3+ blood cell or CD4+ lymphocyte percentage and IL-1ß, IL-2, TNF-α-positive cell percentage in general and on lymphocytes), other factors should be evaluated in future trials, including TGF-β, MMP-1 or MMP-7. Moreover, there was a significantly higher rate of ischemic heart disease in the placebo group, which might have affected the study results. Although some positive trends were observed in the potassium canrenoate group, none of these observations reached statistical significance. Further studies in a larger group of patients are needed to confirm the possible protective effect of potassium canrenoate.

## 4. Materials and Methods

### 4.1. Ethics

This secondary analysis stems from a prospective phase IV randomized placebo-controlled clinical trial (RCT) conducted at University Hospital no. 2 of the Pomeranian Medical University in Szczecin, Poland, spanning from December 2020 to August 2021. The study garnered approval from the Ethics Committee Board at the Pomeranian Medical University in Szczecin, Poland (ICE consent 0012/100/2020, issued on 29 June 2020), and it was duly registered on ClinicalTrials.gov under the identifier NCT04912011.

### 4.2. Study Population

The study enrolled hospitalized patients of all genders, ranging in age from 18 to 90 years, following comprehensive disclosure of study particulars and obtaining their signed informed consent forms. The research adhered to the principles outlined in the Declaration of Helsinki.

**Figure 5:**
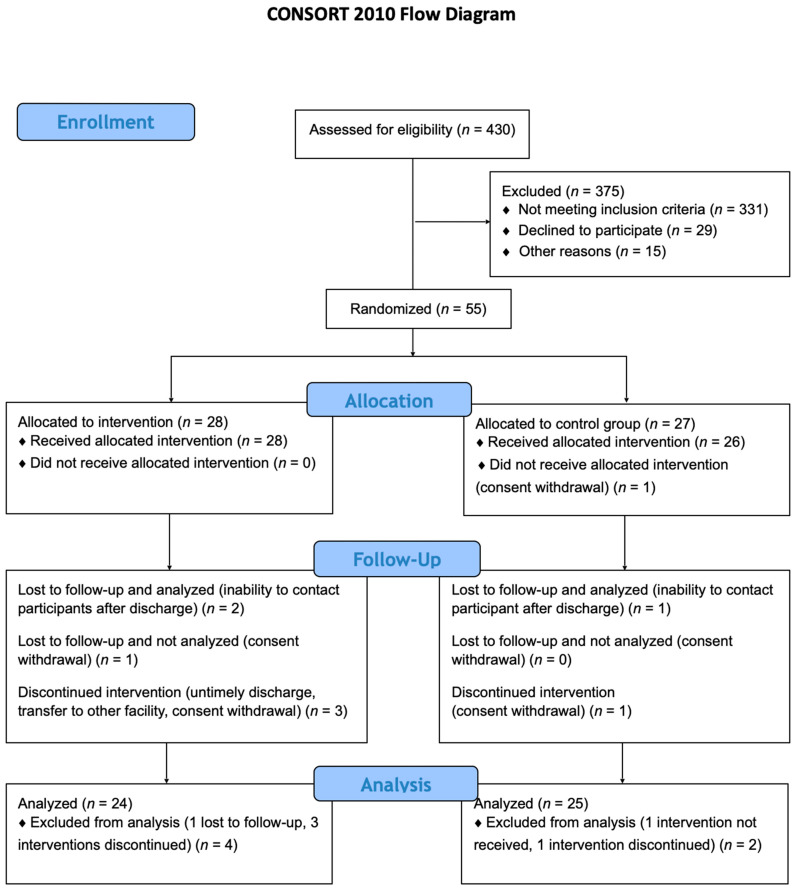
Study flowchart.

In this study, a comprehensive assessment was conducted on a total of 430 patients to determine their eligibility. Following the application of exclusion criteria, 55 patients were selected for randomization, with 49 ultimately included in the final analysis. Among these, 24 were assigned to the intervention group and 25 were allocated to the control group, as illustrated in Figure 5.

### 4.3. Inclusion Criteria

- Individuals of all genders, aged between 18 and 90 years.
- Patients requiring oxygen therapy with a blood oxygen saturation level below 94%.
- Confirmed diagnosis of COVID-19 infection through rt-PCR testing.
- Presence of at least one documented risk factor for heightened COVID-19 mortality, as outlined in current scientific literature, such as smoking, hypertension, diabetes, or cardiovascular disease.
- Well-documented informed consent in accordance with ICH-GCP guidelines and national regulations.

### 4.4. Exclusion Criteria

- Patients with a history of chronic bronchitis, emphysema, interstitial lung disease, or any other preexisting lung conditions.
- Individuals with contraindications for the use of spironolactone.
- Hypersensitivity to spironolactone or any of its components.
- Pregnant individuals (with mandatory pregnancy testing for those of reproductive age) or breastfeeding mothers.
- Patients with mental illness or dementia who are unable to provide informed consent for participation in the study.
- ARDS resulting from another viral infection (testing negative for SARS-CoV-2).
- ARDS caused by other factors or trauma.
- Presence of ionic imbalances, such as hyperkalemia or hyponatremia.
- Adrenal crisis.
- Acute or chronic renal failure, with a creatinine clearance rate below 30 mL/min.
- Anuria.
- Porphyria.
- Chronic use of mineralocorticoid receptor antagonist (MRA) medications from the spironolactone group.

### 4.5. Clinical Experiment Measures

Consecutive patients in the study were randomly assigned to one of two groups using a computer-generated list of numbers. The first group, referred to as the intervention group, received 200 mg of potassium canrenoate (Aldactone) dissolved in 100 mL of 0.9% sodium chloride intravenously twice daily for a duration of 7 days. The second group, known as the placebo group, received 100 mL of 0.9% sodium chloride intravenously twice daily for the same 7-day period.

For the above-mentioned groups of patients, a detailed immunophenotype analysis of blood cell cytometry on day 1 and day 7 of the intervention was performed, including parameters such as T CD4+ lymphocytes, T CD3+ lymphocytes, IL-1ß on lymphocytes, total count of IL-1ß and IL-2 on lymphocytes, and total count of IL-2 and TNF-α on lymphocytes vs. total count of TNF-α. Immunophenotyping results were compared with those from the control group. For flow cytometrical analysis, peripheral blood samples for frequency analysis were collected in ethylenediaminetetraacetic acid-containing tubes. The cells were examined based on unstained control, FMO control, and stained cells with monoclonal antibodies conjugated with fluorescent dyes using 3-color labeling with the following surface antibodies: mouse fluorescein isothiocyanate (FITC)-conjugated anti-human CD4 (clone SK3)/mouse peridinin chlorophyll protein (PerCP)-conjugated anti-CD3 (clone SK7) to determine the proportion of the CD4 + CD3+ T lymphocytes (Becton Dickinson, East Rutherford, NJ, USA), anti-human IL-1β (PE) (BD FastImmune, San Jose, CA, USA), anti-human TNF-α (PE) (FastImmune, San Jose, CA, USA) and anti-human IL-2 (APC) (FastImmune, San Jose, CA, USA). Cells were incubated with an Fc receptor (FcR) blocking reagent (Miltenyi Biotec, Auburn, CA, USA) for 10 min at room temperature to block specific FcR-mediated binding of antibodies. Next, the cells were incubated for 20 min at room temperature with 20 μL of each mAb per sample for 30 min at 4 °C in the dark. After staining of samples with antibodies, cells were treated with lysing solution (Becton Dickinson) and incubated for 15 min at 4 °C in the dark. The cells were then washed twice with phosphate-buffered saline. Cell subsets were detected using cell labeling and gating methods, which start by removing doublets (FSC-A vs. FSC-H), followed by a dot plot, in which lymphocyte populations were defined (FSC vs. SSC). The data were collected on an eight-colour FACSCantoII flow cytometer (BD Biosciences, Franklin Lakes, NJ, USA). Diva software (BD Biosciences, Franklin Lakes, NJ, USA) was used for data analysis, and the percentage of positive cells was recorded. At least 10,000 total events in the physical parameters were acquired for each sample. The percentage of IL-1, IL-2 and TNF-positive cells was calculated from the total number of lymphocytes. Levels of IL-6 have been routinely assessed by hospital laboratory using ELISA as part of the local treatment protocol.

### 4.6. Outcome Measures

#### 4.6.1. Primary Outcome Measures for Primary Analysis

1. Duration of invasive mechanical ventilation via endotracheal intubation or tracheotomy (observation time 30 days).
2. Duration of passive oxygen therapy (observation time 30 days).

#### 4.6.2. Secondary Outcome Measures for Primary Analysis

1. Intensive care unit length of stay (LOS) (time frame 30 days).
2. Total hospital length of stay (LOS) (time frame 90 days).
3. Assessment of the dynamics of recovery of changes in lung ultrasound at 7 days.
4. Assessment of the dynamics of recovery of changes in lung ultrasound at 30 days.
5. Assessment of the dynamics of recovery of changes in chest computed tomography (CT) at 3 months (90 days).
6. Assessment of mortality at 30 days.
7. Assessment of mortality at 90 days.
8. Six-minute walking test (6MWT) at 30 days.
9. Six-minute walking test (6MWT) at 90 days.

#### 4.6.3. Outcome Measures for Secondary Analysis

1. Assessment of the dynamics of change in T CD4+ lymphocyte count at 7 days.
2. Assessment of the dynamics of change in T CD3+ lymphocytes count at 7 days.
3. Assessment of the dynamics of change in IL-1ß on lymphocytes and total count of IL-1ß at 7 days.
4. Assessment of the dynamics of change in IL-2 on lymphocytes and total count of IL-2 at 7 days.
5. Assessment of the dynamics of change in TNF-α on lymphocytes and total count of TNF-α at 7 days.
6. Assessment of the dynamics of change in IL-6 serum level at 7 days.

### 4.7. Statistical Analysis

For the secondary analysis, we used data from the primary analysis. Categorical variables are presented as proportions and were compared using the chi-square test. In the case of small numbers in groups, the Yates correction was applied. Continuous variables are presented as means with standard deviation and medians and first and third quartiles. When assessing statistical significance, Student’s *t*-test or Mann–Whitney *U* for independent samples was used. The Wilcoxon test was used to provide intragroup comparisons between day 1 and day 7. The standard significance level was set at a value of *p* < 0.05. All data were analyzed using the software Statistica 13 (StatSoft, Inc., Tulsa, OK, USA).

For the primary analysis, the sample size was calculated to demonstrate statistical significance of differences in the assessment of the duration of invasive mechanical ventilation via endotracheal intubation or tracheotomy (hours) at 48 h after admission, assuming the standard significance level of the test *p* = 0.05 and power of 0.90. Additionally, it was assumed that the standard deviation (SD) of the length of the duration of invasive mechanical ventilation via endotracheal intubation or tracheotomy time would be 48 h. With the above information taken into the assessment by the statistician, the study size was calculated to include 23 patients per arm, with a minimum total of 46 patients. The research project assumed the number of patients in each group should be 25, because this number was found to be achievable with the incurred costs, study time and availability of patients with predetermined inclusion and exclusion criteria. Participants were randomly divided into one of the two groups (according to the randomization table generated from www.randomiser.com, accessed on 3 December 2020).

## 5. Conclusions

In this secondary analysis of a randomized, placebo-controlled study, the administration of potassium canrenoate to patients with COVID-19-induced pneumonia resulted in an impact on the levels of specific inflammatory markers (including interleukin-6, CD3%, and TNF-α), which could potentially be linked to pulmonary fibrosis. It is worth noting that while some positive trends were observed within the potassium canrenoate group, none of these observations achieved statistical significance. Furthermore, adverse events recorded during the study did not exhibit a statistically significant increase associated with the administration of potassium canrenoate. The findings from this study suggest that any potential benefits of using potassium canrenoate as an anti-inflammatory or anti-fibrotic treatment in COVID-19 patients warrant further investigation.

## Figures and Tables

**Table 1:** Baseline characteristics.

Variables		Potassium Canrenoate (*n* = 24)	Placebo (*n* = 25)	*p*-Value Significance ≤ 0.05
Age [years], mean ± SD; Me		61.54 ± 9.06; 64.00	63.84 ± 14.75; 66.00	0.513
Gender [male], *n* (%)		10 (41.67)	16 (64.00)	0.200
BMI [kg/m^2^], mean ± SD; Me		30.92 ± 4.10; 30.78	30.57 ± 4.63; 29.05	0.780
Smoking, *n* (%)	No	14 (58.33)	12 (48.00)	0.204
	Yes	0 (0.00)	3 (12.00)	
	Quit >1 month	10 (41.67)	10 (40.00)	
Alcohol use, *n* (%)	No	9 (37.50)	7 (29.17)	0.734
	Yes	1 (4.17)	2 (8.33)	
	Occasionally	14 (58.33)	15 (62.50)	
CFS [1,2,3,4,5,6,7], (mean± SD; Me)		3.17 ± 0.70; 3.00	3.76 ± 1.01; 4.00	0.034
Comorbidities				
Arterial hypertension, *n* (%)		15 (62.50)	16 (64.00)	0.851
Ischemic heart disease, *n* (%)		0 (0.00)	7 (28.00)	0.017
Myocardial infarction, *n* (%)		0 (0.00)	4 (16.00)	0.128
Chronic heart failure, *n* (%)		0 (0.00)	3 (12.00)	0.248
Atrial fibrillation, *n* (%)		0 (0.00)	4 (16.00)	0.128
Hypercholesterolemia, *n* (%)		3 (12.50)	8 (32.00)	0.196
Transient ischemic attack, *n* (%)		0 (0.00)	1 (4.00)	0.984
Diabetes, *n* (%)		10 (41.67)	4 (16.00)	0.095
Peripheral vascular disease, *n* (%)		1 (4.17)	5 (20.00)	0.209
Peptic ulcer disease, *n* (%)		0 (0.00)	1 (4.00)	0.984
Thyroid disease, *n* (%)		4 (16.67)	4 (16.00)	0.746
Active neoplasm, *n* (%)		2 (8.33)	2 (8.00)	0.632
Depression, *n* (%)		0 (0.00)	1 (4.00)	0.984

Legend: BMI—body mass index, CFS—Clinical Frailty Scale, Me—median, SD—standard deviation, *p*—statistical significance.

**Table 2:** Laboratory results on day 1.

Variables	Potassium Canrenoate Mean ± SD; Me	Placebo Mean ± SD; Me	*p*-Value Significance ≤ 0.05
White blood cells [G/L]	8.19 ± 3.00; 8.41	7.52 ± 3.06; 7.59	0.440
Neutrophils [G/L]	6.64 ± 2.98; 6.69	6.02 ± 2.84; 5.74	0.461
Lymphocytes [G/L]	1.05 ± 0.30; 0.98	0.95 ± 0.32; 0.90	0.266
Red blood cells [T/L]	4.30 ± 0.41; 4.20	4.20 ± 0.63; 4.27	0.523
Platelets [G/L]	317.04 ± 132.56; 265.00	260.08 ± 93.54; 245.00	0.091
Hemoglobin [mmol/L]	7.97 ± 1.11; 7.90	7.90 ± 0.99; 7.90	0.836
C-reactive protein [mg/dL]	95.58 ± 65.37; 80.14	71.08 ± 44.78; 76.04	0.135
Procalcitonin [ng/mL]	0.23 ± 0.35; 0.09	0.15 ± 0.12; 0.12	0.327
D-dimer [ng/mL]	2329.58 ± 2695.07; 1016.00	1799.32 ± 1902.33; 1158.00	0.432

Legend: Me—median, SD—standard deviation, *p*—statistical significance.

**Table 3:** Laboratory results on day 7.

Variables	Potassium Canrenoate Mean ± SD; Me	Placebo Mean ± SD; Me	*p*-Value Significance ≤ 0.05
White blood cells [G/L]	9.90 ± 4.19; 9.27	10.88 ± 5.77; 10.09	0.512
Neutrophils [G/L]	7.49 ± 4.41; 6.93	8.36 ± 5.81; 6.12	0.574
Lymphocytes [G/L]	1.60 ± 0.62; 1.78	1.57 ± 0.72; 1.44	0.875
Red blood cells [T/L]	4.26 ± 0.43; 4.19	4.31 ± 0.55; 4.33	0.714
Platelets [G/L]	385.87 ± 112.78; 380.00	372.70 ± 119.06; 365.00	0.702
Hemoglobin [mmol/L]	7.89 ± 0.97; 7.80	8.08 ± 0.77; 8.10	0.463
C-reactive protein [mg/dL]	28.35 ± 45.98; 10.50	25.71 ± 41.52; 7.60	0.841
Procalcitonin [ng/mL]	0.20 ± 0.49; 0.06	5.43 ± 23.14; 0.07	0.337
D-dimer [ng/mL]	1782.45 ± 1607.25; 1312.00	1719.48 ± 1826.09; 1105.00	0.903

Legend: Me—median, SD—standard deviation, *p*—statistical significance.

**Table 4:** Immunophenotype analysis on day 1.

Variables	Potassium Canrenoate Mean ± SD; Me	Placebo Mean ± SD; Me	*p*-Value Significance ≤ 0.05
CD4 [%]	23.48 ± 14.64; 22.05	26.20 ± 1.,42; 24.40	0.482
CD3 [%]	12.85 ± 9.46; 11.55	15.66 ± 11.39; 12.65	0.482
IL-1ß on lymphocytes [%]	0.24 ± 0.25; 0.15	0.28 ± 0.43; 0.10	0.605
IL-1ß total [%]	0.68 ± 0.58; 0.45	0.61 ± 0.59; 0.40	0.843
IL-2 on lymphocytes [%]	1.27 ± 1.62; 0.75	1.35 ± 1.31; 1.05	0.475
IL-2 total [%]	4.39 ± 3.76; 3.60	5.24 ± 7.71; 3.75	0.956
TNF-α on lymphocytes [%]	0.15 ± 0.16; 0.10	0.15 ± 0.16; 0.10	0.835
TNF-α total count [%]	0.54 ± 0.45; 0.40	0.53 ± 0.47; 0.35	0.792
IL-6 [pg/mL]	64.97 ± 72.52; 41.00	46.68 ± 56.79; 24.90	0.332

Legend: CD—cluster of differentiation, IL—interleukin, TNF—tumor necrosis factor, Me—median, SD—standard deviation, *p*—statistical significance.

**Table 5:** Immunophenotype on day 7.

Variables	Potassium Canrenoate Mean ± SD; Me	Placebo Mean ± SD; Me	*p*-Value Significance ≤ 0.05
CD4 [%]	31.66 ± 14.92; 28.10	32.92 ± 11.16; 31.10	0.421
CD3 [%]	20.50 ± 14.40; 17.80	21.16 ± 15.37; 16.40	0.950
IL-1ß on lymphocytes [%]	0.43 ± 0.57; 0.20	0.40 ± 0.39; 0.30	0.850
IL-1ß total [%]	1.27 ± 0.83; 1.20	1.16 ± 0.91; 1.00	0.563
IL-2 on lymphocytes [%]	1.42 ± 1.28; 1.20	1.67 ± 0.98; 1.70	0.247
IL-2 total [%]	2.59 ± 1.67; 2.70	2.45 ± 1.94; 2.30	0.554
TNF-α on lymphocytes [%]	0.04 ± 0.12; 0.00	0.02 ± 0.05; 0.00	0.870
TNF-α total count [%]	0.25 ± 0.23; 0.10	0.26 ± 0.31; 0.20	0.841
Interleukin-6 [pg/mL]	24.20 ± 69.38; 5.30	60.56 ± 152.47; 11.00	0.317

Legend: CD—cluster of differentiation, IL—interleukin, TNF—tumor necrosis factor, Me—median, SD—standard deviation, *p*—statistical significance.

**Table 6:** Intragroup comparisons on day 1 versus day 7.

Day 7 versus Day 1	Potassium Canrenoate *p*-Value Significance ≤ 0.05	Placebo *p*-Value Significance ≤ 0.05
CD4 [%]	0.051	0.131
CD3 [%]	0.022	0.181
Interleukin-1ß on lymphocytes [%]	0.177	0.185
Interleukin-1ß total [%]	0.004	0.016
Interleukin-2 on lymphocytes [%]	0.649	0.265
Interleukin-2 total count [%]	0.070	0.040
TNF-α on lymphocytes [%]	0.152	0.003
TNF-α total count [%]	0.031	0.056
Interleukin-6 [pg/mL]	0.006	0.506

Legend: CD—cluster of differentiation, IL—interleukin, TNF—tumor necrosis factor, Me—median, SD—standard deviation, *p*—statistical significance.

## Data Availability

The dataset is not available in a public database due to legal reasons. Anonymous data may be provided by the corresponding author upon a reasonable request from a researcher.
